# Of Passing Interest

**Published:** 1896-01

**Authors:** 


### OF PASSING INTEREST.

Watertown's (New York) second epidemic of typhoid fever has been traced to infected milk, delivered by a dealer who had the disease in his family and had been careless about washing the cans. No less than twenty-five cases occurred on his route, and two or three deaths. When will our cities be preserved from these dangers by a dairy-inspection service by competent veterinarians to guard against these and many other dangers?

The recent stock-show in New York City was a splendid exhibition of stock. The variety and large number of exhibits were well worth a visit by every veterinarian, and must have been a source of great benefit and knowledge to those practising in that city.

Prospective litigation in Pennsylvania by the State Veterinary Examining Board promises to be interesting, bountiful, and we trust successful. Several prospective suits against violators of the act are already under consideration.

A recent suit decided in favor of the plaintiff in the English courts, awarding a verdict of twelve hundred and fifty dollars for depreciation of value of a mare owned by a veterinary surgeon and placed by him in the hands of a trainer, with an express contract that, in the event of any illness or defect of any kind arising in the mare while being trained, the said trainer to immediately inform the owner, who reserved the right to professionally care for her. Negligence was shown in failing to comply with agreement.

Brooklyn has added to her industries an establishment for the preparation of horse-meat for the markets. Bologna-sausages are one of the chief forms of preparation.

Massachusetts seems to be the only State in the Union where the veterinarian is accorded rank in the State militia. Drs. Peters and Osgood, of the U. S. V. M. A., hold place there as commissioned officers. Which State will move next?

A good opening for a competent veterinarian is reported to us at Geneva, Ill. A letter to the Calumet Stock Farm Company, 189 LaSalle Street, Chicago, will afford better information to any of our readers wishing to further investigate this opening.

A large delegation of English stock-raisers recently waited upon President Long, of the Board of Agriculture, imploring him to use his influences in their behalf by preventing further introduction of live-stock from this side of the water. The worn-out cry of contagious pleuro-pneumonia was resurrected, but we feel must have made little impression.

Dr. H. D. Gill, of the New York Veterinary College, was recently interviewed by a reporter of the New York *Recorder* on the scope and character of equine dentistry.

The best method of determining the adulteration of milk seems at present to be in the measurement of butter-fats, and the safest standard one based on the amount of butter-fats.

The methods of conducting the matriculation examinations at the Royal Veterinary Colleges are being strongly criticised, and the subject of severe comment among the several members of the council who direct this work.

Covington, Kentucky, has an Angora cat farm, where a large number of carefully bred cats are raised each year for the increasing demand.

A rabid half-bred Mexican dog recently bit a small boy and a number of pet animals at Columbus, Ohio. Drs. Detmers and Loveberry held autopsy on the dog after its destruction. Cases of this kind have appeared more or less frequently in Columbus, dating back for three years, when a mistaken diagnosis permitted a rabid dog to run at large for a considerable period of time before its destruction.

A recent outbreak of anthrax among London horses was traced to infected oats. A law-suit was the outcome of the results, and damages were awarded by the justice, covering the value of the horses that had died from eating the oats.

Long Island has to-day three horse-meat establishments among her industries.

American beef in London grows in demand daily, and, being cheaper and better than beef grown in England, is largely contributing to its increased sale.

Congressman Hull, of Iowa, has been appointed by Speaker Reed as Chairman of the Military Affairs Committee, which insures to the veterinary profession a warm friend and advocate for recognition in the army.

